# The Institutional Library

**Published:** 1915-04-17

**Authors:** 


### THE INSTITUTIONAL LIBRARY.

### Practical Points on Open-Air Schools.

The Year-book of Open-air Schools and Children's Sanatoria. Edited by T. N. Kelynack, M.D. Volume I., 1915. (London: John Bale, Sons and Danielsson, Ltd. Price 7s. 6d.)

Open-air schools and sanatoria for children have abundantly justified themselves, although the movement is still in its initial stages. From playground classes to a fully equipped residential open-air school is a fairly long step, but the latter class of institution must inevitably become available for the large numbers of tuberculous children now being "discovered" if real permanent benefit is to accrue to the next generation of citizens. Forty hours' weekly attendance out of 168 at a so-called open-air school is not enough to counteract faulty home conditions, and the good work of the daytime is too often undone at night. In this country there are many difficulties to contend against in establishing suitable schools, but the lay mind is undoubtedly being prepared as the outcome of the work of school medical officers and tuberculosis officers for the establishment on an extended scale of open-air schools or schools of recovery. Those persons working towards this object will find in this year-book much information of an encouraging nature. If there are sceptical individuals who doubt the value of such institutions they will surely revise their opinions after studying this work. As a companion volume to the "Tuberculosis Year-book," which was published last year, this latest proof of the industry of the editor, Dr. Kelynack, will meet with acceptance, if it does not even eclipse the former year-book as a ready reference work on the subject of tuberculosis in children and the scholastic problems pertinent thereto. The large number of original communications by medical authorities are supplemented by articles and plans by architects and by ladies and gentlemen actively engaged in teaching or other educational work. The keynote of this volume is practical information, and not the least useful section is that giving notes on appliances, preparations, and equipment suitable for cases undergoing open-air treatment. A feature of the book which will at once commend itself is the very large number of illustrations; the practical utility of this feature cannot be overrated, and almost by itself justifies the publication of this year-book. From these pictures and plans an excellent idea can be gained of what has been already done under very varying conditions to meet the need for open-air schools and children's sanatoria. The section of the work dealing with the schemes now under way in different parts of the country is far from being complete; only a few districts receive mention at present, though these are representative. In the future, no doubt, this section will be expanded till it forms a fairly complete survey of the kingdom and a comparative study of work being done in this field. It is hardly necessary to point out what a valuable asset such a work of reference would be. Of children's sanatoria, special hospitals, homes, and colonies there is given a good selection, many of which are described in detail, but some receive very meagre notice. In the section relating to open-air schools there are some excellent descriptions and illustrations of individual schools. At the end of the book is to be found a handy directory of tuberculosis officers, school medical officers and their assistants, with a list of dental clinics, special schools, etc., in England and Scotland. This book should inevitably increase in usefulness as time goes on, till it becomes indispensable to all medico-educational workers.

### The Theory and Practice of After-Care.

The Inevitable Complement. The Care and After-Care of Consumptives. By Harold Vallow, M.D. (London: John Bale, Sons and Danielsson, Ltd. 1915. Price 1s. 6d.)

The author in this little book has expounded his views on the way in which After-Care Committees may be formed, and how they should direct their efforts to ensuring the continued well-being of consumptives who have been or are under treatment. The book is evidently meant for the lay voluntary worker, in spite of the preface; for there is much therein which could only be described as well-worn platitudes from the viewpoint of the tuberculosis officer. Dr. Vallow gives as his model the After-Care Committee which was formed in Bradford last year, and describes some of its duties. There is nothing very novel to be found in this little book; After-Care Committees have been working in connection with certain tuberculosis dispensaries for some years. Yet in these pages we have a useful guide which might with advantage be perused by intending helpers. By dint of subdivision and repetition the matter has been spread over sixty-six pages, a space that seems excessive, having regard to the amount of information conveyed. A table on page 39, relating to sleeping arrangements, is not very illuminating, and calls for reconstruction. One of the appendices consists of an article on visiting by the Secretary of the Bradford Charity Organisation Society, and another gives a list of suggested occupations for consumptives, by McGaw.

### The Medical Woman's Opportunity.

English Medical Women: Glimpses of their Work in Peace and War. By A. H. Bennett, with a Preface by Stephen Paget, F.R.C.S. With Three Illustrations. (Sir Isaac Pitman and Sons, Ltd. 1915. Price 3s. 6d.)

This pleasant and enthusiastic volume has been written by Miss Bennett "in order that the history of the entrance of modern women into the medical profession may be more widely known." As a matter of fact, their work is now having the best and most conceivably wide advertisement, but that is no reason why the general public should not be informed of how it came about that the fierce old prejudice that medicine was an improper profession for a womanly woman was at last broken down, or of the battle at Edinburgh and the early pioneers, three of whose photographs adorn this volume. The author's style of writing is rather that of the nervous layman entering a hospital for the first time, and rather frightened at imaginary bogeys of etiquette and what not, but that has not prevented her from giving a readable account of the general lines of the work now being carried on at the New Hospital, in the Harrow Road, and in South London. No doubt the chapters dealing with the work of medical women in the war will be turned to eagerly, and the subject might even have been dealt with much more fully if proper facilities had been obtained and the scope of the book had admitted. Still, the result is a breezy account which should be instructive to the public which it seeks to reach, and even institutional workers may learn, perhaps with surprise, of several new institutions under women medical management which are mentioned in it. An instance of the general extension of medical work on the part of women is afforded in the last chapter, which gives an account of the Medico-Psychological Clinic which was opened last autumn in London. But the general public will especially appreciate the glimpses of military surgery performed by women at their hospitals at the seat of war.

